# Imbalance Between Oxidative Stress and Growth Factors in Human High Myopia

**DOI:** 10.3389/fphys.2020.00463

**Published:** 2020-05-14

**Authors:** Salvador Mérida, Vincent M. Villar, Amparo Navea, Carmen Desco, María Sancho-Tello, Cristina Peris, Francisco Bosch-Morell

**Affiliations:** ^1^Departamento de Ciencias Biomédicas, Instituto de Ciencias Biomédicas, Universidad Cardenal Herrera-CEU, CEU Universities, Valencia, Spain; ^2^Departamento de Cirugía, Facultad de Ciencias de la Salud, Universidad Cardenal Herrera-CEU, CEU Universities, Valencia, Spain; ^3^Department of Medical Ophtalmology, Fundación para el Fomento de la Investigación Sanitaria y Biomédica (FISABIO) de la Comunitat Valenciana, Valencia, Spain; ^4^Department of Pathology, Universitat de València, Valencia, Spain

**Keywords:** high myopia, oxidative stress, VEGF, HGF, total nitrates, TAC

## Abstract

Myopia is one of the commonest eye pathologies that could affect 2.56 billion people by 2020. Today high myopia is a leading cause of blindness worldwide due to associated ocular illness. Nevertheless, the cellular bases for these diseases to develop are unclear in many areas. We conducted a prospective study of oxidative stress and growth factors in human myopic and non myopic eyes in an attempt to increase our understanding of the underlying physiopathological conditions to adequately early diagnose, prevent and treat the retina problem that derives from myopia. Aqueous humor samples were obtained from 41 patients being operated for cataracts in our hospital. Axial length, refractive status and complete ophthalmologic examination were recorded. The VEGF and HGF levels were determined by an ELISA kit. Total antioxidant capacity and total nitrites/nitrate levels were established with a lab kit. We show for the first time an increase in the total nitrite levels in high myopia. We also propose for the first time the concurrence of three factors: myopia, oxidative stress, and oxidative stress together with growth factors in the same group of patients. In this way, it would not be accurate to envision high myopia as a type of normal myopia, but one with more diopters or longer axial length.

## Introduction

Human myopia is one of the most common eye disorders of significant world public health concern (Young, 2009). Estimations reveal that 49.8 and 9.8% of the people in the world will be affected by myopia and high myopia, respectively, by 2050 (Holden et al., 2016). Similarly, visual impairment associated with myopic macular degeneration will grow to reach 55.7 million people (Fricke et al., 2018). In Europe, myopia prevalence is about 24.3% with an age-standardized prevalence of 30.6% (Williams et al., 2015). In past decades, age-standardized myopia prevalence has increased from 17.8% among those born between 1910 and 1939 to 23.5% in those born between 1940 and 1979. The increase in myopia prevalence is even more marked in young European adults, with a prevalence of 47.2% (Williams et al., 2015). Myopia is one of the main causes of visual impairment, together with macular degeneration, glaucoma, macular hemorrhage and cataracts (Saw et al., 2005). For all these conditions, oxidative stress has been observed to play a relevant role (Bosch-Morell et al., 2015). Genetic and environmental factors have also been associated with this disease (Lin et al., 2009; Wojciechowski, 2011).

This is a twofold problem: on the one hand, myopia is an optical problem that leads to poor focusing due to a mismatch between lenses and eyeball axial length; on the other hand, patients can be predisposed to suffer other eye diseases, like glaucoma, macular hemorrhage, retinal detachment and cataracts (Saw et al., 2005). The best way to deal with this twofold problem is to approach it by considering that two classical myopia categories exist according to different prognosis types. Normal myopia (NM) is moderate and usually associated with values below 6 negative diopters (D) or with eyeball axial length under 26 mm. In the present study these cases are considered low myopia (LM); any other values above these values are considered high myopia (HM), also known as pathological, degenerative or malign myopia. Eyeball length in HM patients becomes longer during their lifetime, which eventually leads to complete blindness. Nowadays, HM is a leading cause of blindness worldwide because of associated eye diseases like retinal detachment, glaucoma, macular choroidal degeneration and cataracts (Young, 2009). Together with continuous eyeball axial length prolongation and reduced scleral thickness in functional HM deterioration, atrophy takes place in retinal pigment epithelium (RPE) and choroids, which leads to oxidative stress in a hypoxia environment (Wu et al., 2018; Zhu et al., 2018).

Several studies show the importance of genetics and HM, namely growth factors (Lin et al., 2009; Wojciechowski, 2011). Transforming Growth Factor (TGF-β), basic Fibroblast Growth Factor (bFGF), and Insulin-like Growth Factor (IGF) (Guo et al., 2015) are all factors that focus on eye growth control deregulation. Sun et al. (2019) showed that genetic variants of cytokine Fibroblast Growth Factor 10 (FGF10) are associated with susceptibility to myopia and HM in young children. Similarly, the connective tissue growth factor (CTGF) level is also elevated in highly myopic eyes (Ding et al., 2019). Hepatocyte Growth Factor (HGF) centers on the neuroprotective role and may be involved in idiopathic epiretinal membrane growth (Ding et al., 2019), while Vascular Endothelial Growth Factor (VEGF) focuses on myopic choroidal neovascularization (CNV) (Bosch-Morell et al., 2015).

It is well-known that a disproportionate production of reactive oxygen species (ROS) disrupts the imbalance between antioxidant defenses and free radical production, which plays an important role in the physiopathology of many other human eye diseases, including myopia (Nita and Grzybowski, 2016). Our research group proposed oxidative damage playing an essential role in LM, and especially in HM, by showing statistically significant differences linked to lipid peroxidation between LM and HM patients (Bosch-Morell et al., 1996).

The retina is not only one of the main oxygen-consuming tissues, but it is also one of the tissues with the most marked oxidative environments in the human body (Lamb and Pugh, 2006; Marquioni-Ramella and Suburo, 2015). Any vascular alteration would modify oxygen supply and could lead to transitory hypoxia because eye vascularization supplies the retina with high partial oxygen pressure. Oxidative stress in the retina may trigger a cascade of events, including loss of RPE and photoreceptors (Juel et al., 2013). Oxidative stress, which is generated in hypoxic situations of the myopic retina, is crucial in the retina for its good blood flow and photic oxidative injury (Lange et al., 2008) and high polyunsaturated fatty acids content (Guo et al., 2015; Riddell et al., 2016). A recent work by our research group showed significantly different metabolite signatures related to nitric oxide synthase (NOS) and oxidative stress in the aqueous humor of HM patients (Barbas-Bernardos et al., 2016).

Previous works have suggested that HGF and VEGF might play a relevant role in the physiopathological molecular mechanisms present in myopia disease (Veerappan et al., 2010; Shin et al., 2012). The present work was a prospective study of the VEGF and HGF levels. It determined oxidative stress in the aqueous humor of patients with and without myopia. Its goal was to identify if there was a direct connection between oxidative damage and different growth factors.

## Materials and Methods

### Selection of Patients and Myopia Grade Classification

Aqueous humor samples were collected from 41 eyes of 41 patients about to undergo eye surgery at the FISABIO Eye Hospital. Twenty myopic patients and a control group composed of aqueous samples from 21 patients were selected, who were age-matched with the myopic group. All the participants in the study were Caucasian and presented cataract eyes with nuclear color and opalescence, cortical or posterior subcapsular cataract 2–3, according to the Lens Opacities Classification System III (Chylack et al., 1993). Patients’ medical histories were widely reviewed and discussed with the patients. Any other eye or systemic disease was considered an exclusion criterion. The study protocol complied with the Declaration of Helsinki. It was reviewed and approved by the Ethics Committee of the FISABIO Eye Hospital. Informed consent was obtained from all the subjects who participated in the study.

IOLmaster^®^ interferometry axial length was obtained by preoperative ocular refraction. A complete ophthalmologic examination (best corrected visual acuity, intraocular pressure, anterior and posterior segment examinations) was performed with all the patients. Axial length was measured by the Cooper Vision Ultrascan Digital B 2000 system, specifically in the autofreeze mode. Measurements were taken until three acceptable A-scan traces were recorded per eye by the same researcher. The only traces to be excluded were those presenting off-axis alignment, i.e., those with echoes. The accepted traces were the A-Scans with retinal echoes at low amplification and a strong lens. A slit-lamp was used with a special adapter to mount the applanation-type ultrasound probe to minimize variability in corneal indentation, which can be associated with a handheld A-Scan (Raj et al., 1998).

Patients were divided into three groups: the Control group (C), consisting of emmetropic individuals, astigmatism below 2.75 D or hypermetropia under +1.75 D; the LM group, with axial length below 26 mm; the HM group, with axial length exceeding 26 mm (Flores-Moreno et al., 2013; Hosoda et al., 2018). As all our patients had cataracts, refractive status was not used to classify so as to avoid any bias caused by index myopia (Díez-Ajenjo et al., 2015).

### Aqueous Humor Sample Collection

After preparing the surgical field with 5% povidone iodine, sterile draping and placement of speculum, aqueous humor samples were collected from all the patients under sterile conditions using a 30-gauge needle connected to a 1cc sterile syringe prior to cataract surgery. Undiluted aqueous samples (0.05 ml) were collected from each patient. Samples were placed inside sterile Eppendorf tubes that were stored immediately at −80°C.

### Biochemical Analysis

The VEGF and HGF levels were determined with a commercially available multiplexed sandwich enzyme-linked immunoabsorbent assay (ELISA) kit (Searchlight Human Angiogenesis Array; Pierce Biotechnology, Inc., Woburn, MA, United States), in which assays were run following a classic dot sandwich ELISA protocol using biotin-streptavidin-horseradish peroxidase amplification and chemiluminescence detection. Assays were carried out using 25 μl aqueous humor volumes (the sample size range recommended by the kit went from 10 to 50 μl/well).

The antioxidant-oxidant status was evaluated in the aqueous humor samples collected from both the patients and healthy controls. Serum samples were assayed for total antioxidant capacity (TAC). TAC was measured using a lab kit (Cayman Chemical, Ann Arbor, MI, United States) following the manufacturer’s instructions. This assay is based on the ability of antioxidants in human aqueous humor samples to inhibit the oxidation of 2,2′-azino-bis-(3-ethylbenzthiazoline-6-sulfonate) (ABTS) by metmyoglobin. The amount of oxidized ABTS is detected by measuring absorbance at 750 nm. Each human aqueous humor sample was measured in duplicate and TAC levels were always expressed as μmol/ml.

Total nitrites (stable end product of nitric oxide) and nitrate levels were determined in the aqueous humor samples of both the patient and control groups by a commercially available kit (Parameter, Total Nitric Oxide and Nitrate/Nitrite Assay, R&D Systems, Minneapolis, MN, United States). This assay quantifies nitric oxide concentrations based on the enzymatic transformation of nitrate into nitrite by nitrate reductase. This reaction is monitored by the colorimetric detection of nitrite as an azo dye product of the Griess Reaction. Aqueous humor nitrate/nitrite levels were expressed as μmol/l. The purpose of measuring these levels was twofold: on the one hand, as an indicator of nitric oxide production which is important as such as an eye modulator; on the other hand, as a marker of oxidative stress (d’Azy et al., 2016; Perdices et al., 2018).

### Statistical Analysis

Data are shown as mean ± SE. Statistical analyses were performed with the commercially available software IBM SPSS version 24.0 (IBM Corp. Released 2016. IBM SPSS Statistics for Windows, Version 24.0. Armonk, NY: IBM Corp., United States) and GraphPad Prism version 7.04 for Windows (GraphPad Software, La Jolla, CA, United States).

Normality was tested (*p* > 0.05) by the Kolmogorov–Smirnov test. Statistical significance for the intergroup difference was assessed by a one-way analysis of variance with a *post hoc* analysis for normal variables, or by the Kruskal–Wallis test for the non normal variables, as appropriate. The ANOVA of the data found by the Levene test was performed by taking the Tukey test as a *post hoc* test whenever homogeneity in data variances was indicated (*p* < 0.05). Similarly, the Kruskal-Wallis test was performed for the non parametric variables when the homogeneity in data variances was present. Additionally, comparisons among groups were made by the false discovery rate-adjusted *P*-value (FDR) < 0.05.

To examine the strength of the association between two variables, correlations were examined by Pearson’s or Spearman’s tests.

Statistical significance was set at the *p* ≤ 0.01 and *p* ≤ 0.05 levels. Table 1 summarizes the study groups.

**TABLE 1:** Summary of the study groups.

	**Control (C)**	**Low Myopia (LM)**	**High Myopia (HM)**
N	21	12	8
Age (years)	58.6 ± 14.3	69.7 ± 11.6	64.2 ± 12.2
Eye axial length (mm)	22.7 ± 1.4	23.7 ± 1.2	29.8 ± 2.7^#^
Refractive error (D)	−0.1 ± 1.1	−3.4 ± 3.8	−14.9 ± 4.6^#^

**The refractive error and eye axial length values are expressed as mean ± SE. ^#^p < 0.01 vs. C and LM groups.**

## Results

### HM Patients Present Altered HGF and VEGF Levels in Aqueous Humor

**FIGURE 1:**
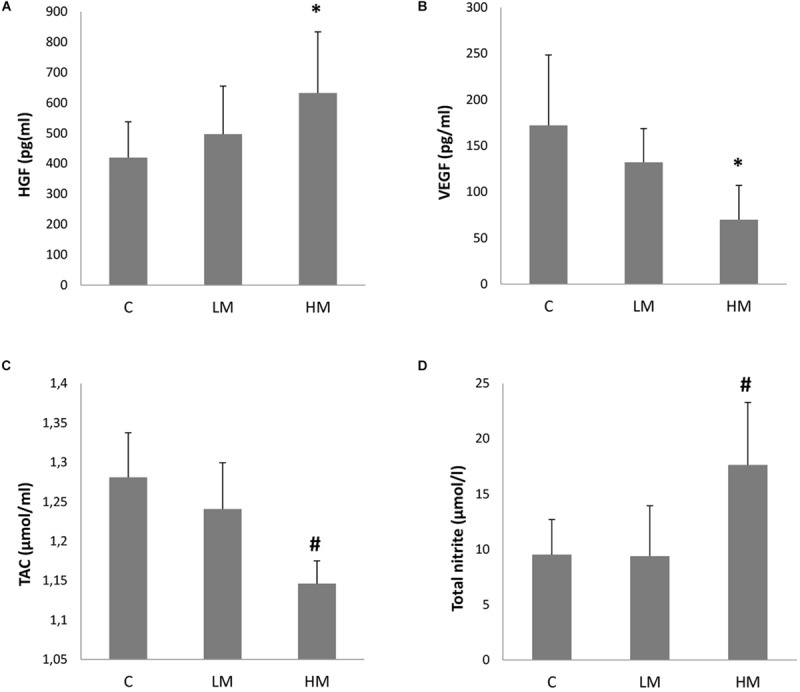
Growth factors levels (**A**, HGF and **B**, VEGF) and oxidative stress parameters (**C**, TAC and **D**, total nitrite/nitrate) in the collected aqueous humor of the Control group (*n* = 21), the Low Myopia group (*n* = 12) and the High Myopia group (*n* = 8). Each value represents the mean ± SE. *p < 0.01 vs. the C group; ^#^*p* < 0.01 vs. the C and LM groups.

The myopia patients showed an increased tendency for higher aqueous humor HGF levels (Figure 1A). Therefore, the aqueous humor HGF levels significantly increased and reached levels of 632.5 ± 201.2 pg/ml in the eyes of these HM patients (*p* < 0.01). However, no significant differences were observed between the C (420.1 ± 117.5 pg/ml) and LM (496.9 ± 158.3 pg/ml) groups (p > 0.05).

The aqueous humor VEGF level was 172.1 ± 76.4 (mean ± SD) pg/ml in the eyes of the C group. The myopia patients presented lower aqueous humor VEGF values (Figure 1B). The HM patients obtained significantly lower levels for this growth factor with levels of 69.9 ± 37.2 pg/ml (*p* < 0.01 vs. the C group). The levels in the LM patients (132.1 ± 36.6 pg/ml) were also lower, but no significant differences were found with the C group (*p* > 0.05).

### Myopia Increases Oxidative Stress in Aqueous Humor

The oxidative stress parameter of the total antioxidant capacity in aqueous humor is shown in Figure 1C. The TAC value was 1.28 ± 0.06 (mean ± SD) μmol/ml in the eyes of the C group. The myopia patients presented lower TAC values in both groups, and this value in the LM patients was 1.24 ± 0.06 μmol/ml (*p* > 0.05) and 1.15 ± 0.03 μmol/ml in the HM patients (*p* < 0.01 vs. the C and LM groups).

The oxidative stress parameter of total nitrite/nitrate in aqueous humor is shown in Figure 1D. No significant difference was observed between the C (9.53 ± 3.17 μmol/l) and LM (9.39 ± 4.55 μmol/l) groups (*p* > 0.05). However, the HM group levels significantly increased with values of 17.63 ± 5.66 μmol/l (*p* < 0.01 vs. the C and LM groups).

### Measured Oxidative Stress Parameters Significantly Correlate With VEGF and HGF Levels in All Patients

As explained above, total nitrite and TAC were the oxidative stress parameters measured in the aqueous humor, which gave values that correlated significantly with the measured growth factor levels (*p* < 0.05).

**FIGURE 2:**
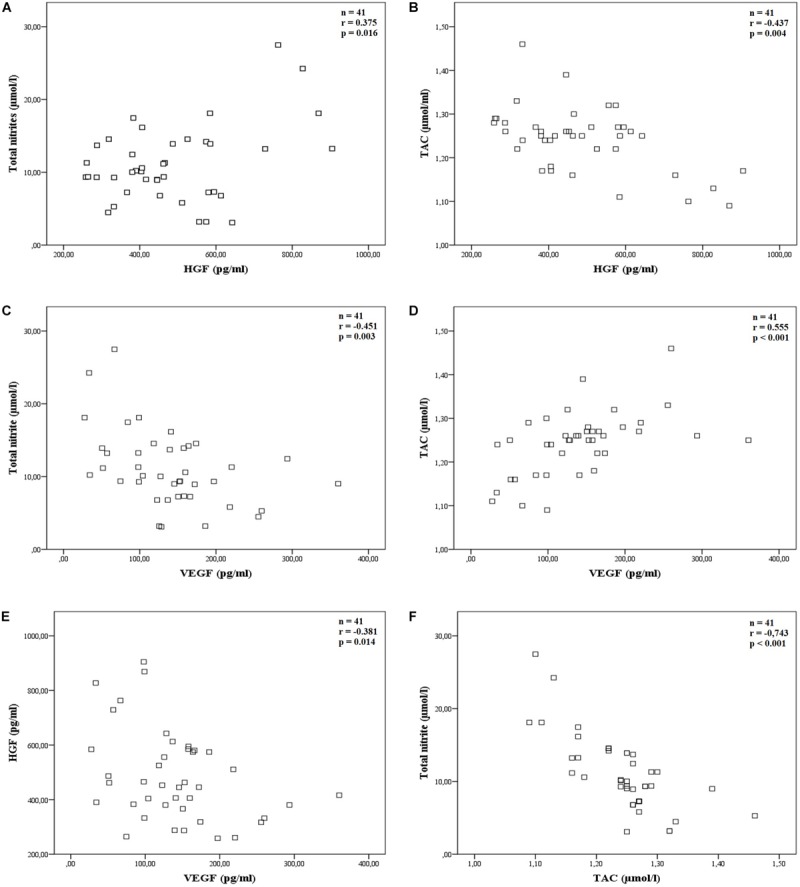
Correlations of the main studied parameters. The scatter plot showing Pearson’s or Spearman’s correlation between: **(A)** total nitrite and HGF, *r* = 0.375, *p* = 0.016 (*n* = 41); **(B)** TAC and HGF, *r* = −0.437, *p* = 0.004 (*n* = 41); **(C)** total nitrite and VEGF, *r* = −0.451, *p* = 0.003 (*n* = 41); **(D)** TAC and VEGF, *r* = 0.555, *p* < 0.001 (*n* = 41); **(E)** HGF and VEGF, *r* = −0.381, *p* = 0.014 (*n* = 41) and **(F)** total nitrite and TAC, *r* = −0.743, *p* < 0.001 (*n* = 41).

HGF was better correlated with TAC than with total nitrite. Figure 2 presents the Scatter plots and shows the Pearson’s correlation between total nitrite and HGF with an *r*-value of 0.375, *p* = 0.016, *n* = 41 (Figure 2A), and Spearman’s correlation between TAC and HGF with an *r-*value of −0.437, *p* = 0.004, *n* = 41 (Figure 2B).

Similarly, VEGF was better correlated with TAC than with total nitrite parameters. Pearson’s correlation between total nitrite and VEGF had an *r*-value of −0.451, *p* = 0.003, *n* = 41 (Figure 2C), and Spearman’s correlation between TAC and VEGF had an *r*-value of 0.555, *p* < 0.001, *n* = 41 (Figure 2D).

Finally, Pearson’s correlation between HGF and VEGF had an *r*-value of −0.381, *p* = 0.014, *n* = 41 (Figure 2E) and total nitrite and TAC clearly correlated well (Spearman’s correlation, *r* = −0.743, *p* < 0.001, *n* = 41, Figure 2F).

### Correlations With Myopic Parameter Eye Axial Length

Spearman’s correlations between the classification of eyes based on refractive eye status in D and on eye axial length in mm with the measured variables, such as eye axial length in mm, HGF levels (pg/ml), VEGF levels (pg/ml), TAC (μmol/l) and total nitrite (μmol/l), were calculated. Spearman’s correlation coefficients were calculated using the data collected exclusively from the 20 myopia patients (Table 2). HGF showed no significant correlation with either the refractive error classification (*r* = 0.323, *p* > 0.05) or the eye axial length classification (*r* = 0.283, *p* > 0.05). It was noteworthy that the oxidative stress parameters presented a similar correlation with the refractive error classification (TAC, *r* = −0.763, *p* < 0.001; total nitrite, *r* = 0.658, *p* = 0.002) than with the eye axial length classification (TAC, *r* = −0.719, *p* < 0.001; total nitrite, *r* = 0.629, *p* = 0.003). However, the VEGF data indicated a better correlation with the eye axial length classification (*r* = −0.827, *p* < 0.001) than with the refractive error classification (*r* = −0.635, *p* = 0.003).

**TABLE 2:** Spearman’s correlation coefficient between patients’ refractive and biometric parameters with the measured variables.

	**Eye axial length (mm)**	**Refractive error (D)**
HGF (pg/ml)	0.283	0.323
VEGF (pg/ml)	−0.827**	−0.635**
TAC (μmol/ml)	−0.719**	−0.763**
Total nitrite (μmol/l)	0.629**	0.658**
Refractive error (D)	0.865**	

*Coefficients were calculated using the data collected exclusively from the patients (n = 20) classified by diopters or eye axial length (mm). Significance is denoted by* *p < 0.05 and **p < 0.01.

No statistically significant differences were found in the mean ages (*p* < 0.05) of the LM [mean ± SD; 69.7 ± 11.6 years (range, 55–85)], HM [mean ± SD; 64.2 ± 12.2 years (range, 46–80)] and C [58.6 ± 11.2 years (range, 44–76)] groups. Table 2 provides information on the patient group about refractive power in diopters and eye axial length in mm.

## Discussion

In the present study, both the measured oxidative stress parameters (i.e., TAC and total nitrite/nitrates) significantly altered in the aqueous humor of the HM patients (Figures 1C,D). The TAC values tended to lower, which was indirectly proportional to the myopia grade classification. Therefore, the aqueous humor TAC levels significantly dropped in the HM group (*p* < 0.01). A correlation between the TAC levels and refractive error (*r* = -0.763, *p* < 0.001) or eye axial length (*r* = -0.719, *p* < 0.001) in the myopia patients was also found. Depletion of TAC as an indicator of the overall capability to neutralize oxidative stress suggests excess ROS, which may cause oxidative damage to deoxyribonucleic acid, proteins and lipids (Niki, 2010). In the myopic eye, different biometric alterations take place, such as axial axis. Under these conditions, retina vascularization would reduce and alter ocular pulse amplitude (Shih et al., 1991), to consequently generate transitory hypoxia states that are concomitant with the imbalance of cellular pro-oxidant-antioxidant species (Bosch-Morell et al., 2015). Under these conditions, ROS production is related to mitochondrial stimulation (Abramov et al., 2007), xanthine activation (Rieger et al., 2002) and calcium-dependent NADPH oxidases (Matsuo et al., 2009). Thus under pathological conditions, NADPH oxidases promote the release of ROS from endothelial cells (Eelen et al., 2018). Likewise, the up-regulation of ROS may lead to a more marked stimulation of nuclear factor-κB (NF-κB) and, consequently, to more nitric oxide (NO) and inflammatory cytokines being released (Roy et al., 2017).

Some works have also previously linked oxidative stress and myopia. In 1989, Simonelli et al. (1989) proposed the idea that lipid peroxidation could be involved in cataracts developing in patients with severe myopia and diabetes. Later this idea was confirmed because greater glutathione oxidation occurs in severe myopia, which is frequently related to glaucoma and maculopathy in crystalline and vitreous humor (Micelli-Ferrari et al., 1996). In patients with myopia, our laboratory found a relation between their diopters and the lipid peroxidation levels in the subretinal fluid of patients with retinal detachment, as well as significant differences between LM and HM patients (Bosch-Morell et al., 1996). In contrast with other studies, Kim et al. (2016) found that levels of 8-Hydroxydeoxyguanosine, a product of oxidative DNA damage, was lower in the aqueous humor of highly myopic eyes compared to that in control eyes. However, the same author acknowledges that there are other studies, like that by Micelli-Ferrari et al. (1996), which report quite the opposite results. Furthermore, Zhu et al. (2018) showed the down-regulation of antioxidant genes in HM eyes via DNA hypermethylation. Our study coincides with most previous ones and, for the first time, proposes herein the co-existence between three factors: myopia, oxidative stress, and oxidative stress together with growth factors in the same group of patients.

We show, for the first time, in HM an increase in total nitrite levels (Figure 1D, *p* < 0.01). Total nitrate is a stable end product of nitric oxide. This parameter has been traditionally used as an oxidative stress marker, which could suggest that nitric oxide, specifically through peroxynitrite, could be considered one of the origins of oxidative stress damage and reduced antioxidant capacity in HM, as described in our studies and in some others. NO is produced by different isoforms of NOS and plays a relevant role in many intricate cellular routes in human eye tissues (Becquet et al., 1997), which are included in both the vascular tone control of the basal membrane in the retina and flicker-induced vasodilation of the retina (Dorner et al., 2003; Wu et al., 2012). Neuronal and endothelial NOS (nNOS and eNOS, respectively) need calcium for activation, but cytokine-inducible NOS (iNOS) display calcium-independent activity and may generate NO in large amounts under pathological conditions (Cavet et al., 2014). All three NOS isozymes are expressed in the human choroid (Park et al., 1994). Even though nitrite has long since been considered a final metabolite of nitric oxide, this role has been recently revised in the literature and could act as a storage pool of NO, which can be reduced to nitric oxide under hypoxic conditions (Shiva, 2013). This makes our work even more relevant because nitrite increases. Furthermore, nitric oxide is an eye modulator in developing myopia, such as ocular growth, retinal vascular development, intraocular pressure regulation, and so on. Nevertheless it is not easy to interpret these results given the ambivalent role of nitric oxide as a neurodegenerative/neuroprotective agent (Contestabile et al., 2003; Pannu and Singh, 2006), and it is even more complicated due to the different isoforms of NOS that might be differently involved in retina and choroid neovascularization (Ando et al., 2002), or in ocular growth of several animal models of myopia (Nickla et al., 2006).

No significant difference appeared in the total nitrite/nitrate levels between the C and LM groups (Figure 1D, *p* > 0.05), but a large significant difference was found in the total nitrite/nitrate levels of the HM group (Figure 1D, *p* < 0.01). A correlation between the total nitrite levels and refractive error (*r* = 0.658, *p* = 0.002) or eye axial length (*r* = 0.629, *p* = 0.003) was also found in the myopia patients. It is known that oxidative damage associated with hypoxic myopia can disturb the neuromodulation that both NO and dopamine present during eye growth and development (Jiang et al., 2014). NOS uses L-arginine as a substrate, as well as reduced nicotinamide-adenine-dinucleotide phosphate (NADPH) and molecular oxygen as co-substrates (Eelen et al., 2018). Therefore, these results agree with previous works. Thus in the aqueous humor of HM patients, our research group found two metabolites, namely arginine and citrulline (Barbas-Bernardos et al., 2016), which are related through NOS regulation (Hattenbach et al., 2000; Eelen et al., 2018). Moreover, in different animal models of myopia, such as guinea pig and chicken, increased NOS activity has been observed along with the concomitant up-regulation of cGMP in posterior ocular tissues (Fujikado et al., 1997; Nickla et al., 2006; Wu et al., 2007). In fact Nickla and coworkers have suggested that a relevant NOS isoform, namely the neuronal one, is implicated in choroidal thickness changes in response to myopic defocus in chicken (Nickla et al., 2009). Similarly, Wen and collaborators have reported that the expressions of NMDAR1 and nNOS mRNA in the retina of guinea pigs with form-deprivation myopia are enhanced (Wen et al., 2015).

Excess nitrite/nitrate levels reveal potential cytotoxic effects. Excess NO and ROS production may promote the formation of other reactive nitrogen oxides by, thus, reacting with O_2_^–^, which may generate peroxynitrite (ONOO^–^) that can trigger oxidation damage, nitration and S-nitrosilation of proteins, lipids and DNA (Villanueva and Giulivi, 2010; Förstermann and Sessa, 2012). Similarly, NO may also react with either cytochrome c oxidase or soluble guanylate cyclase to produce nitrative stress (Villanueva and Giulivi, 2010). NO also increases mitochondria-derived ROS production (Kietadisorn et al., 2012).

The TAC levels in this study showed a decreasing tendency in myopic groups, with more marked decreases in the HM group (Figure 1C, *p* < 0.01), which is an optimal environment where several factors can promote different pathways, such as NF-κB, and cause oxidative/peroxynitrite stress in HM. Moreover, eNOS uncoupling leads to greater ROS production by changing it from an NO-producing enzyme to an enzyme that generates O_2_^–^. Therefore in vascular pathologies, the uncoupling of eNOS has been linked to the oxidation of BH4, an essential cofactor required for normal eNOS activity (Kietadisorn et al., 2012). Instead, diminished enzymatic antioxidant via hypermethylation may also be present (Zhu et al., 2018).

Our present findings about VEGF are similar to those obtained by previous works (Shin et al., 2012; Yuan et al., 2019). VEGF is expressed by RPE during choroidal vascular growth and in fully grown tissue (Saint-Geniez et al., 2006). It is known that aqueous levels of VEGF and other vasoactive molecules, such as IL-6, are related to vitreous levels (Funatsu et al., 2005; Noma et al., 2008). Several studies have shown relevant visual amelioration in the myopic choroidal neovascularization of patients who have been administered an intravitreal anti-VEGF injection (Willis et al., 2017). In animal models of superoxide dismutase 1-deficient mice, oxidative stress has stimulated VEGF-induced subretinal neovascularization (Dong et al., 2009). Similarly, 4-hydroxynonenal stimulated VEGF expression in human RPE cells has been reported to act as a strong lipid peroxidation agent (Ayalasomayajula and Kompella, 2002). However, our results found that myopia patients showed low VEGF levels, which strongly and significantly lowered in the HM group (Figure 1B), and a high negative Spearman’s correlation was also observed between the VEGF levels and eye axial length (*r* = -0.827, *p* < 0.001). Previous works have suggested that lower VEGF levels in myopic eyes may be due to dilution by a larger intraocular volume (Hu et al., 2015), which is responsible for myopic retinal degeneration (Yuan et al., 2019). Interestingly, Yuan et al. (2019) have suggested that low-grade intraocular inflammation might play a relevant role in HM and myopic retinopathy. All our results agree with this approximation.

The VEGF that derives from RPE is involved in natural choroidal neovascularization regulation (Marneros et al., 2005), and takes care of retina microvasculature by two different ways: VEGFR2-facilitated vasculotrophism and by regulating complement proteins and, consequently, protecting against potential complement-mediated damage (Keir et al., 2017). Nevertheless, many experimental data have related oxidative stress, mainly chronic intracellular oxidative stress, to the persistent danger of RPE cells integrity (Plafker et al., 2012) via NF-kB signaling (AnandBabu et al., 2019). HM is related to RPE thinning (Fujiwara et al., 2009). Therefore, RPE cells display multiple degenerative alterations in HM, and even the suppression of the function of RPE cells may lead to VEGF depletion (Shin et al., 2012). Another important growth factor to be considered is pigment epithelium-derived factor (PEDF), an anti-angiogenic and neuroprotective factor that is also produced and secreted by both RPE cells and retinal ganglion cells (Miyazaki et al., 2011; Miyake et al., 2013). In this way, our results also suggest that the VEGF/PEDF ratio may be disturbed in RPE cells. Lastly, the relation between nitric oxide and VEGF is particularly relevant. Nitric oxide is a key molecule in the signaling pathway for VEGF (Thachil, 2011) and it decreases the VEGF induced by hypoxia through the dependent mechanism of cGMP (Ghiso et al., 1999). At the same time, anti-VEGF drugs protect from the oxidative stress modulated by the release of NO, apoptosis and autophagy in human RPE cells (De Cillà et al., 2017). Although it is very complicated to interpret an increase of nitrite in HM, it is clear that it plays an essential role that needs to be further studied.

HGF is a relevant cytokine that has been found in different ocular tissues, including RPE, cornea and choroidal endothelial cells, which can produce endothelial development and neovascularization via the c-MET signaling pathway (Organ and Tsao, 2011). It is up-regulated under hypoxia, helps retinal vascular permeability (Vasir et al., 2000; Clermont et al., 2006) and can increase eNOS activity by the phosphoinositide 3-kinase/Akt pathway, which leads to vasodilation in the eye microvasculature (Makondo et al., 2003; Uruno et al., 2004). HGF also up-regulates the expression of matrix metalloproteinase 2 in scleral fibroblasts of myopic guinea pigs, which is related to the greater degradation of the extracellular matrix and axial elongation (Li et al., 2014).

Our results are consistent with these preceding works because we found HGF to also be overexpressed (Figure 1A). The HGF gene has been also associated with myopia (Han et al., 2006; Veerappan et al., 2010). In fact genetic variation in HGF has been found in children in Singapore, which has been associated with the retinal arteriolar diameter that, in turn, has been related to the pathogenesis of eye disease in these children (Khor et al., 2010). Furthermore, HGF can play an essential role in preventing oxidative stress and may, thus, act as a therapeutic tool to protect against myopic hypoxia.

## Conclusion

In the present study, oxidative stress parameters TAC and total nitrite/nitrates were found to significantly alter in the aqueous humor of HM patients. For the first time, an increase in nitrites (nitric oxide) in HM was demonstrated. A strong correlation was also found among these parameters and myopia development. Oxidative stress may, thus, help to explain altered regulatory pathways in myopia. Some previous scientific evidence relates myopia to oxidative stress and myopia with growth factors, VEGF and HGF separately. The present study proposes for the first time a correlation among the three factors: myopia, oxidative stress, and oxidative stress together and the growth factors in the same group of patients. In other words, we reveal a significant correlation between oxidative stress with both VEGF and HGF in the same group of myopia patients.

It is not accurate to envision HM as a type of normal myopia, but one with more diopters or longer axial length. Our research centers on the molecular pathways in each myopia patient group. They qualitatively differ from one another if we bear in mind that HM is one of the most serious problems that ophthalmology faces and is currently growing worldwide. Further studies are needed to evaluate the role of these correlations in the exact mechanisms implicated in HM-related complications.

## Data Availability Statement

The datasets generated for this study are available on request to the corresponding author.

## Ethics Statement

The studies involving human participants were reviewed and approved by Ethics Committee of the FISABIO Eye Hospital. The patients/participants provided their written informed consent to participate in this study.

## Author Contributions

SM, AN, and FB-M conceived and designed the experiments. MS-T and FB-M performed the experiments. SM and CP analyzed the data. SM, VV, AN, CD, and FB-M wrote the manuscript. EG-G performed final revision and corrections of the manuscript.

## Conflict of Interest

The authors declare that the research was conducted in the absence of any commercial or financial relationships that could be construed as a potential conflict of interest.

The reviewers declared a shared affiliation with the handling Editor at the time of review.
